# The autism-related gene SNRPN regulates cortical and spine development via controlling nuclear receptor Nr4a1

**DOI:** 10.1038/srep29878

**Published:** 2016-07-19

**Authors:** Huiping Li, Pingping Zhao, Qiong Xu, Shifang Shan, Chunchun Hu, Zilong Qiu, Xiu Xu

**Affiliations:** 1 Department of Child Health Care, Children’s Hospital of Fudan University, 399 Wanyuan Road, Shanghai 201102, China; 2 Institute of Neuroscience, Key Laboratory of Primate Neurobiology, CAS Center for Excellence in Brain Science and Intelligence Technology, Shanghai Institutes for Biological Sciences, Chinese Academy of Sciences, Shanghai 200031, China

## Abstract

The small nuclear ribonucleoprotein polypeptide N (*SNRPN*) gene, encoding the RNA-associated SmN protein, duplications or deletions of which are strongly associated with neurodevelopmental disabilities. *SNRPN*-coding protein is highly expressed in the brain. However, the role of SNRPN protein in neural development remains largely unknown. Here we showed that the expression of SNRPN increased markedly during postnatal brain development. Overexpression or knockdown of SNRPN in cortical neurons impaired neurite outgrowth, neuron migration, and the distribution of dendritic spines. We found that SNRPN regulated the expression level of Nr4a1, a critical nuclear receptor during neural development, in cultured primary cortical neurons. The abnormal spine development caused by SNRPN overexpression could be fully rescued by Nr4a1 co-expression. Importantly, we found that either knockdown of Nr4a1 or 3, 3′- Diindolylmethane (DIM), an Nr4a1 antagonist, were able to rescue the effects of SNRPN knockdown on neurite outgrowth of embryonic cortical neurons, providing the potential therapeutic methods for SNRPN deletion disorders. We thus concluded that maintaining the proper level of SNRPN is critical in cortical neurodevelopment. Finally, Nr4a1 may serve as a potential drug target for SNRPN-related neurodevelopmental disabilities, including Prader-Willi syndrome (PWS) and autism spectrum disorders (ASDs).

The small nuclear ribonucleoprotein polypeptide N (*SNRPN*) gene, encoding the RNA-binding SmN protein, is located within chromosome 15q11-q13 in the region associated with various neurodevelopmental disabilities such as Prader-Willi syndrome (PWS), Angelman syndrome (AS) and autism spectrum disorders (ASDs)^1^,^2^,^3^. These neurodevelopmental disorders are often associated with various degrees of autistic behavior and learning disabilities. The *SNRPN* gene is imprinted with preferential expression from the paternal chromosome^4^,^5^ and usually transcribed as the downstream gene of the bicistronic SNURF-SNRPN mRNA. The SmN protein is a member of the small nuclear ribonucleic particle SMB/SMN family, which is involved in pre-mRNA splicing^6^,^7^. The SMB/SMN family proteins were originally defined on the basis of autoantibodies present in sera from patients with systemic lupus erythematosus (SLE)^8^. Differential methylation patterns of the *SNRPN* gene have been reported to reflect multiple types of cancer, indicating that SNRPN may be critical in tumorigenesis^9^,^10^,^11^.

Interestingly, SNRPN is expressed in a tissue-specific manner, with the highest expression levels in the adult brain and heart^12^. Many distinct alternative splicing events take place in neuronal tissue, suggesting the critical role of SmN in regulating alternative splicing. Previous studies proposed that SmN was involved in catalyzing CGRP-specific splicing^13^. However, certain discrepancies showed that SmN was involved in other neuron specific splicing events and that neither required or sufficient for regulating alternative splicing of the calcitonin/CGRP transcript^14^. According to the study by Huntriss and colleagues^15^, there are no significant changes in select brain-specific alternative splicing events in SNRPN knockout mice. Thus, the function of SNRPN in neural development remains to be further addressed.

A recent study by Lee and coworkers found that ectopic expression of SmN was associated with increased expression of 4 genes and decreased expression of 23 genes, as detected by massive parallel sequencing in the HeLa cell line with an inducible expression system for SmN, suggesting that the important role of SmN in regulating gene expression^16^. The nuclear receptor subfamily 4, group A, member 1 (Nr4a1) (also known as NGFI-B/NUR77), an activity-dependent gene encoding a nuclear receptor, was included in the 23 SmN-mediated downregulated genes^16^. Nr4a1 belongs to a family of orphan nuclear receptors (Nr4a1, Nr4a2 and Nr4a3) that play an important role in maintaining cellular homeostasis. These factors have been suggested to be potential drug targets for treating multiple diseases, including cancer^17^,^18^.

Nr4a1 was recently found to have crucial effects on neural development and plasticity, in addition to affecting metabolic, and immune functions^19^. In the central nervous system, Nr4a1 expression is induced by learning tasks, such as contextual fear conditioning^20^, and has been linked to synaptic remodeling^21^,^22^. Chen and colleagues^23^ reported that Nr4a1 overexpression resulted in elimination of the majority of spines via transcriptional regulation of the actin cytoskeleton. Furthermore, Nr4a1 knockdown increased the density of spines specifically at the distal ends of dendrites, suggesting that endogenous Nr4a1 prevents abnormal spine clustering. Spine density is usually correlated with the strength of excitatory synaptic transmission, which is crucial for brain development and cognitive functions, such as learning and memory.

Copy number variation on human chromosome 15q11–q13 is one of the most frequent chromosomal aberrations in ASDs^2^. A paternally inherited duplication of 15q11–q13 with an extra copy of *SNRPN* gene is associated with development/speech delays, mental retardation, and ASDs features^24^,^25^,^26^,^27^. A mouse model with paternal duplication of this region also showed abnormal phenotypes compared with wild-type mice, such as impaired social interaction, abnormal development of ultrasonic vocalization, and resistance to change behavior and anxiety^28^,^29^. The neural mechanisms underlying behavioral abnormalities remain largely unclear. The objective of this study was to determine the function of SNRPN in cortical neurodevelopment. The results demonstrate that abnormal expression of SNRPN impairs neurological function through regulating Nr4a1 and thus Nr4a1 represents a potential therapeutic target for SNRPN-associated diseases.

## Results

### Expression of SNRPN in the developing brain

**Figure 1:**
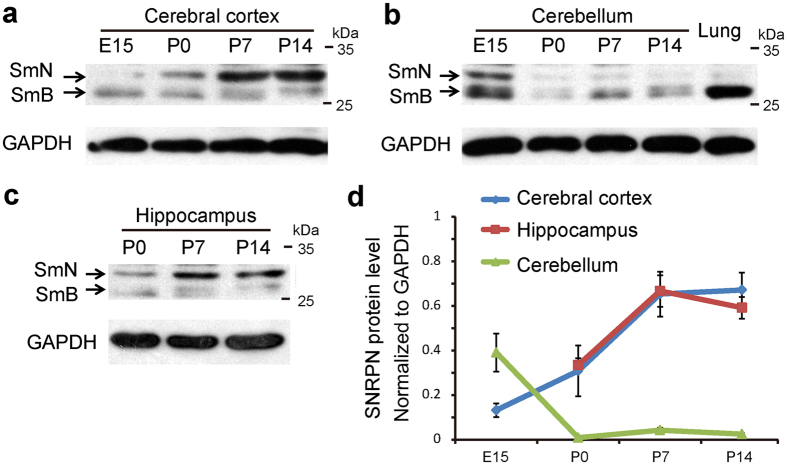
Expression of SmN in the developing cerebral cortex, hippocampus, and cerebellum. (**a**) Homogenates of mouse cerebral cortex at different developmental stages (E15, P0, P7, and P14) were subjected to western blot with SmN and GAPDH antibodies. (**b**) The expression of SmN in mouse cerebellum at E15, P0, P7, and P14. (**c**) The expression of SmN in mouse hippocampus at P0, P7, and P14. (**d**) Quantification of the expression of SmN in the developing brain. Error bars represent ± SEM. Each experiment was repeated independently for at least 3 times.

To examine the expression of SNPRN in the developing brain, we carried out western blotting experiments with brain samples at different developmental stages (embryonic day 15 (E15), postnatal day 0 (P0), P7, and P14). A 28 kDa band and 29 kDa band were detected. According to previous work, the 28 kDa band represented SmB, which is also highly expressed in lung. SmB exhibits 92.5% homology at the amino acid level to SmN and therefore is detectable with the SmN antibody^14^. SmB is a ubiquitous splicing protein, which is almost entirely replaced by SmN in neurons. The 29 kDa band represented SmN. SmN expressed at a low level in the embryonic cerebral cortex and increased approximately 5-fold during brain development (Fig. 1a,d). In the hippocampus, the expression profile of SNRPN was similar to that seen in the cerebral cortex (Fig. 1c,d). However, there was a gradual decrease in expression of SmN in the cerebellum from the embryonic stage to the neonatal stage (Fig. 1b,d).

### Impact of SNRPN on the development of neurites and radial migration

**Figure 2:**
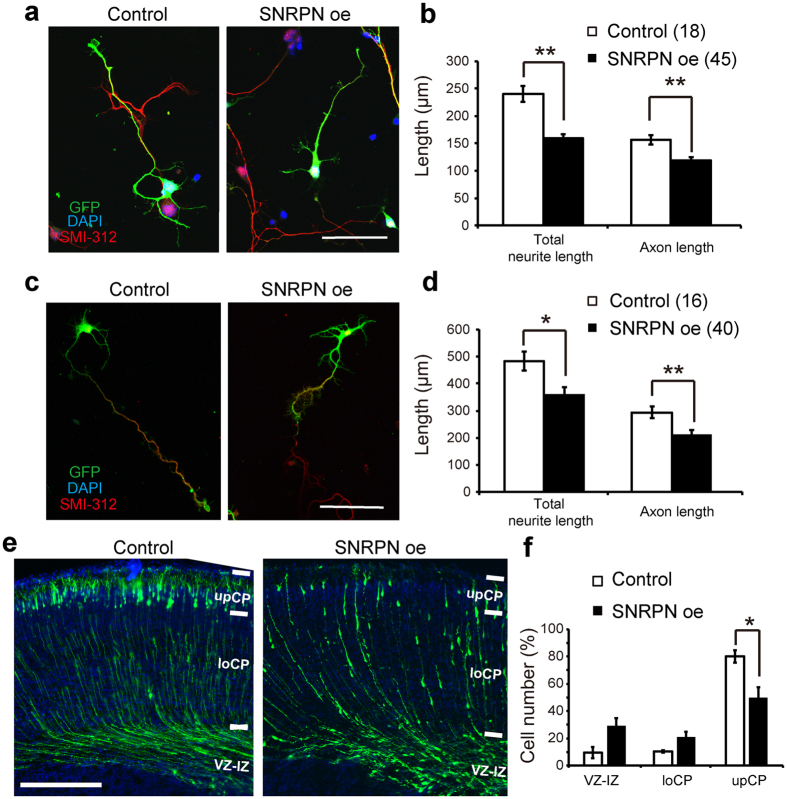
SNRPN overexpression at the embryonic stage impacted the length of neurites and radial migration in the cerebral cortex. (**a**) HA-SNRPN-transfected cortical neurons at E15 cultured for 72 h *in vitro* were stained for GFP (green), DAPI (blue) and SMI-312 (red). The pCAG-IRES-GFP vector was used as a control. Scale bar, 50 μm. (**b**) Quantification of total neurite length and axon length. (**c,d**) Representative images and quantification of neurite length from cortical neurons transfected with control and SNRPN plasmids, DIV7. Scale bar, 100 μm. Numbers in brackets are numbers of neurons analyzed in each group. (**e**) Effects of upregulation of SNRPN in mouse cortical neurons by *IUE* with plasmids coding for HA-SNRPN. Coronal brain sections at P1 were stained with anti-GFP (green) and DAPI (blue). Scale bar, 250 μm. (**f**) Percentage of labeled cells in VZ-IZ, LoCP, and UpCP to total cells electroporated with control (pCAG-IRES-GFP vector) or HA-SNRPN constructs. Data are from at least 3 independent *IUE* experiments. Error bars, SEM. *P < 0.05, **P < 0.01 (Student’s *t*-test).

We next investigated the role of SNRPN in the development of cortical neurons. First, we found that transfection of cultured cortical neurons with a plasmid encoding HA-tagged SNRPN for 72 h resulted in a reduction in the total length of neurites and axons compared with cultures transfected with control (pCAG-IRES-GFP) vector (Fig. 2a,b). The effect of SNRPN to neuritic growth persisted at day *in vitro* 7 (DIV7) (Fig. 2c,d). The identity of the longest neurite as an axon was determined by staining with the axonal marker SMI-312. However, overexpression of SNRPN had no effect on neuronal polarity as we found that most neurons with SNRPN overexpression contained one single axon (Data not shown).

Second, we applied *IUE* to deliver SNRPN with EYFP into cortical progenitor cells at E14.5 and examined the role of SNRPN in cortical migration. We compared the percentage of cell number in the ventricular zone to intermediate zone (VZ-IZ), lower cortical plate (LoCP), and upper cortical plate (UpCP) between neurons carrying an empty vector and overexpressing SNRPN at P1 (Fig. 2e,f). We found that the VZ-IZ and LoCP contained more neurons electroporated with the SNRPN sequence in SNRPN expressing neurons, comparing to control neurons. Percentage of cell number in UpCP of SNRPN expressing neurons was significantly decreased (Fig. 2e,f). These data suggest that overexpression of SNRPN delays radial migration of cerebral cortex neurons.

### SNRPN modulates the expression level of Nr4a1

**Figure 3:**
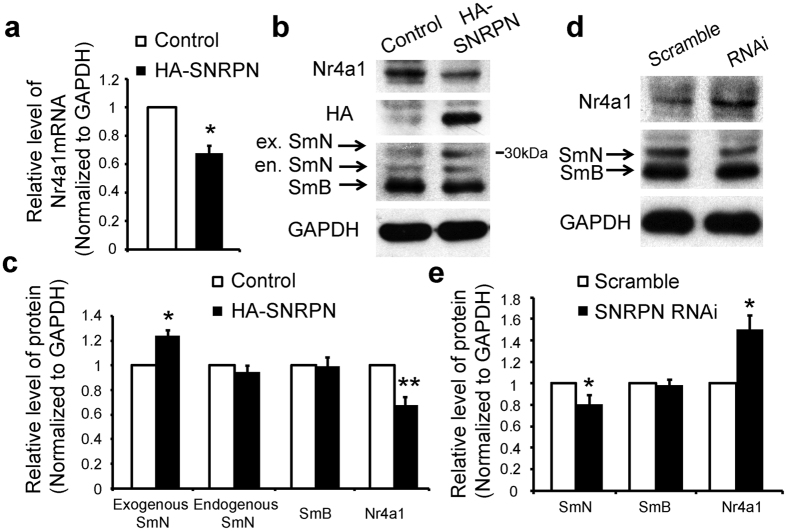
SNRPN downregulated the expression of Nr4a1. (**a**) E15 cortical neurons were electroporated with control (pCAG-IRES-GFP) vector or HA-SNRPN plasmid. 72 h later, Nr4a1 mRNA was quantified by qPCR. (**b**) Western blot showed the change in the Nr4a1 protein level by SNRPN overexpression in cultured cortical neurons. Anti-HA antibody was used to detect the effect of HA-tagged SNRPN plasmid. Anti-SNRPN antibody was used to examine exogenous and endogenous SNRPN proteins, including SmN and SmB. The band about 30 kDa represents exogenous HA-tagged SmN. (**c**) Quantification of the expression of exogenous SmN, endogenous SmN, SmB, and Nr4a1 in cultured cortical neurons transfected with control and HA-SNRPN plasmids. (**d**) Regulation of Nr4a1 protein level by SNRPN shRNA and control constructs (Scramble) in cultured cortical neurons. SmN antibody was used to detect the effect of SNRPN shRNA. (**e**) Quantification of the expression of SmN, SmB, and Nr4a1 in cultured cortical neurons transfected with scramble and SNRPN RNAi plasmids. Data were normalized to GAPDH. The RNA or protein level of the control was set as ‘1’. Error bars, SEM. *P < 0.05, **P < 0.01 (Student’s *t*-test). Each experiment was repeated independently for at least 3 times.

A recent study found that ectopic expression of SmN decreased Nr4a1 expression in Hela cell lines^16^. To determine whether SNRPN modulates the expression of Nr4a1 in neurons, cultured cortical neurons transfected with vector and HA-tagged SNRPN plasmid at DIV3 were processed for qPCR and western blot. Molecular mass of HA-tagged SmN is 29 kD (SmN) plus around 1 kD (HA). We found a higher band about 30 kDa (slightly bigger molecular weight comparing to untagged SmN) was detected in HA-SNRPN expressing neurons, representing exogenous HA-tagged SmN (Fig. 3b). Whereas, protein levels of endogenous SmN and SmB were not affected (Fig. 3c). We found that the mRNA and protein levels of Nr4a1 were significantly down-regulated upon SNRPN expression (Fig. 3a–c). In addition, we designed a shRNA against mouse SNRPN, and it was shown to be effective in suppressing SNRPN expression in cultured primary cortical neurons (Fig. 3d,e). We measured the protein level of Nr4a1 and found that it was significantly increased in neurons with SNRPN knockdown (Fig. 3d,e). Of note, SNRPN knockdown had no effects on the protein level of SmB (Fig. 3d,e).

### SNRPN regulates the density and distribution of dendritic spines via Nr4a1

**Figure 4:**
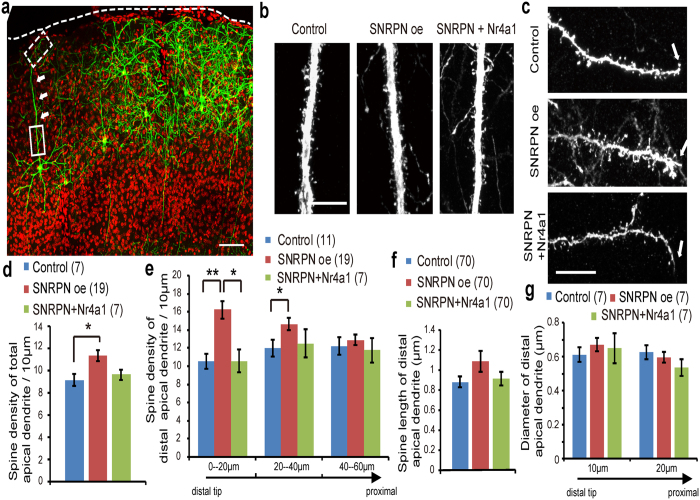
Effect of SNRPN overexpression on the density and distribution of spines in embryonic cortical neurons. (**a**) A large view of Layer 2/3 pyramidal neuron in *IUE* experiments. The white dash line illustrates apical surfaces. The neurons on the edge of densely-labeled neuron group with no surrounding neurons containing GFP were included in the statistics. White arrows indicate apical dendrites from the soma to the distal end. The box with solid lines illustrates a proximal apical dendrite. The box with dashed lines illustrates a distal apical dendrite. Scale bar: 100 μm. (**b**) Representative proximal apical dendrites of P21 mouse cortical neurons transfected with EYFP plus pCAG-IRES-GFP vector (left), SNRPN (middle), and SNRPN with Nr4a1 (right) construct by *IUE*. Scale bar: 10 μm. (**c**) Representative images of spines at the distal end of apical dendrites from pyramidal neurons transfected with EYFP plus pCAG-IRES-GFP vector (top), SNRPN construct (middle), or SNRPN + Nr4a1 construct (bottom) by *IUE*. White arrows indicate the distal tip of the dendrite. Scale bar: 10 μm. (**d**) Quantification of the spine density of apical dendrites from the proximal to distal ends. (**e**) Quantification of the spine density of the distal apical dendrites (binned in 20 μm segments from the tip) shown in (**c**). (**f**) Quantification of spine length of distal apical dendrites (0–20 μm from the tip) in neurons transfected with control, SNRPN, and SNRPN + Nr4al plasmids by *IUE*. (**g**) Quantification of diameter of distal apical dendrites (at 10 μm from the tip, and 20 μm from the tip) in neurons transfected with control, SNRPN, and SNRPN + Nr4al plasmids by *IUE*. Numbers in brackets are the numbers of neurons or spines analyzed in each group. Error bars, SEM. *P < 0.05, **P < 0.01 (Student’s *t*-test).

Because Nr4a1 is a key component of the regulation of the density and distribution of dendritic spines, we next examined whether SNRPN may regulate spine development. We first used *IUE* with plasmids coding for HA-tagged SNRPN and empty vector as control, together with EYFP in cortical progenitor cells in the ventricular zone (VZ) of mouse cortex at E14.5. The longest dendrites were designated as apical dendrites. We counted spine number of the total length of apical dendrite completely showed from soma to distal tips, and the distal ends of apical dendrite (0–60 μm from dendrite tip, binned in 20 μm segments from the tip), respectively (Fig. 4a). At P21, spine density was mildly increased in total apical dendrites of cortical neurons overexpressing SNRPN (Fig. 4b,d). Remarkably, the density of spines increased significantly at the distal ends of apical dendrites in neurons transfected with SNRPN (Fig. 4c,e).

We then tested whether the increased number of distal dendritic spines was mediated by Nr4a1. We transfected SNRPN with Nr4a1 into neurons by *IUE* and measured dendritic spine density in apical dendrites. We found that in neurons co-transfected with SNRPN and Nr4a1 plasmids (see Supplementary Fig. S1), spine density was similar to control (Fig. 4b,d). Notably, the abnormal increase in spine density at the distal ends of dendrites was fully rescued by co-expression of an Nr4a1 cDNA (Fig. 4c,e). Our findings are in line with a previous report stating that Nr4a1 knockdown results in clustered spines at the distal ends of dendrites and further indicates that SNRPN regulates spine development through controlling Nr4a1 expression^23^.

We also measured the spine length of the distal ends of apical dendrite, which were not statistically different upon SNRPN expression (Fig. 4f). The expression of SNRPN also did not impact the diameter of distal apical dendrites notably (Fig. 4g). There evidences showed that SNRPN specifically regulated the distal spine development, rather than general aspects of spine morphology.

### Abnormalities caused by *loss-of-function* of SNRPN was rescued by Nr4a1 knockdown or antagonist

**Figure 5:**
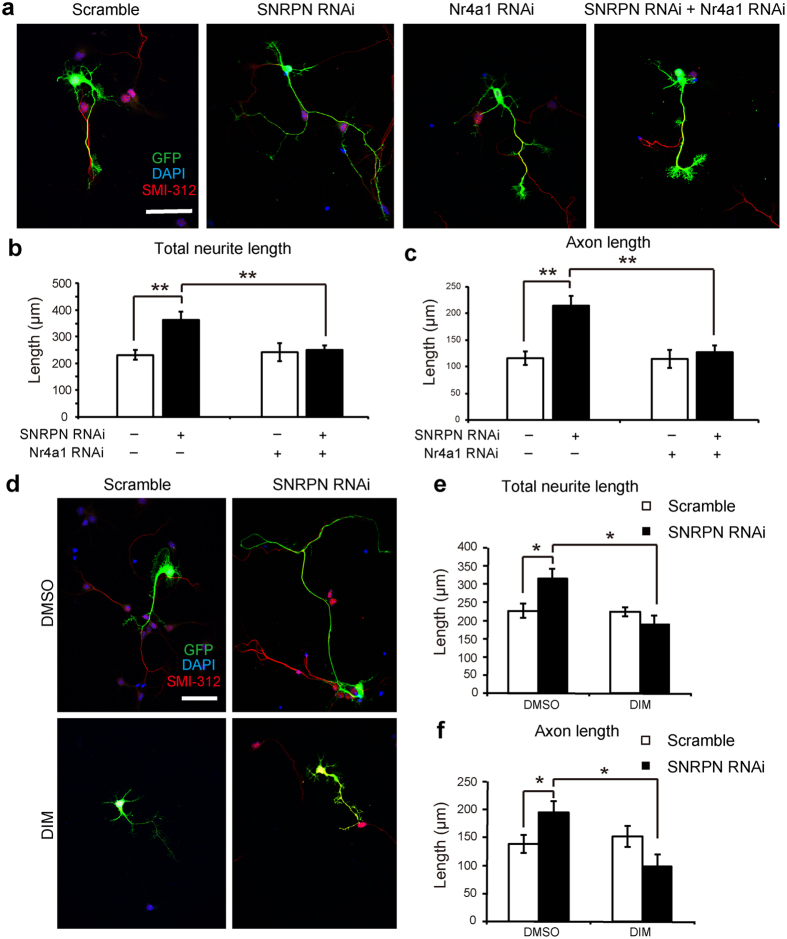
Nr4a1 knockdown rescued the effect of SNRPN RNAi. (**a**) Images showed the cultured cortical neurons transfected with scramble, SNRPN RNAi, Nr4a1 RNAi, and SNRPN RNAi + Nr4a1 RNAi plasmids for 72 h. Cells were stained for GFP (green), DAPI (blue) and SMI-312 (red). Scale bar, 50 μm. (**b,c**) Average length of total neurites and axons of GFP^ + ^neurons. Numbers of neurons analyzed in each group: 18 for scramble group, 18 for SNRPN RNAi group, 18 for Nr4a1 group, 40 for SNRPN RNAi + Nr4a1 RNAi group. (**d**) Embryonic cortical neurons transfected with SNRPN RNAi and scramble plasmids were treated by DIM and DMSO for 48 h *in vitro*, respectively. Representative images show cells stained for GFP (green), DAPI (blue) and SMI-312 (red). Scale bar, 50 μm. (**e,f**) Average length of total neurites and axons of GFP^+^ neurons. Numbers of neurons analyzed in each group: 22 for scramble + DMSO group, 33 for SNRPN RNAi + DMSO group, 8 for scramble + DIM group, 15 for SNRPN RNAi + DIM group; Error bars, SEM. *P < 0.05, **P < 0.01 (Student’s *t*-test).

Consistently, we found that neurites and axons significantly increased upon SNRPN knockdown by RNAi in embryonic cortical neurons cultures (Fig. 5a–c). Finally, we asked whether inhibition of Nr4a1 may be able to rescue the effect of SNRPN knockdown. Interestingly, we found that co-transfected with Nr4a1 RNAi completely rescued the increased length of neurites and axons of embryonic cortical neurons transfected with SNRPN RNAi and did not impact neurotic length in the control group, confirming that SNRPN regulates neuritic growth via Nr4a1 (Fig. 5a–c).

Because 3, 3′-Diindolylmethane (DIM) is a well-established Nr4a1 antagonist and one of the major bioactive components of crucifers, we further examined whether DIM may have effects on defects caused by SNRPN knockdown^30^. DIM analogs bind Nr4a1 at the ligand binding pocket and function as Nr4a1 antagonists in cancer^18^,^31^. Strikingly, we found that DIM treatment was able to fully rescue the outgrowth caused by SNRPN knockdown, similar with Nr4a1 RNAi, thus further providing a pharmacological way to rescue defects of SNRPN *loss-of-function* (Fig. 5d–f). This evidence indicates that Nr4a1 is responsible for defects caused by SNRPN knockdown and that pharmacological inhibition of Nr4a1 is a potential therapeutic target for SNRPN-related brain disorders.

## Discussion

The expression of SNRPN in the embryonic cerebral cortex and hippocampus gradually increases during brain development, consistent with previous work by Grimaldi K. *et al*.^6^ that revealed that during rodent brain development, the levels of SmN rise such that SmN replaces SmB as the predominant protein in the adult brain. SmN exhibits 92.5% homology to SmB at the amino acid level but is encoded by a distinct gene^14^. At present, it is unclear why such homologous small nuclear ribonucleoprotein particle (snRNP) proteins exist and why SmN replaces SmB in neurons. However, the balance between SmN and SmB must be involved in brain development. Abnormal expression of SmN in the embryonic stage results in abnormal development of neuron morphology and function.

In our study, we found that SNRPN overexpression shortened the length of neurites and delayed radial migration in the cerebral cortex. SNRPN knockdown increased the length of neurites. The change in neuronal morphology was subtle. These results perhaps explain why the paternal inherited 15q11-13 duplication can give rise to developmental delays and autistic behavior, whereas maternal duplications exhibit reduced penetrance^32^. The neuropsychological symptoms of PWS (paternal inherited 15q11-13 deletion) are less severe than those of AS (maternal inherited 15q11-13 deletion).

To further examine the potential mechanisms of SNRPN, we focused on SNRPN modulation of Nr4a1 expression. Our idea resulted from the massive parallel sequence data from a HeLa cell line with an inducible expression system for SNRPN^16^. In the 27 genes affected by ectopic expression of SNRPN, Nr4a1 is known as a synaptic regulator, and Nr4a1 mRNA levels decreased in the pilot test of neurons transfected with SNRPN cDNA. There were no significant changes in SLC2A3 or SYDE1 (data not show), which were also critical for neuron development. Western blotting was used to determine that SNRPN downregulated expression of Nr4a1.

The function of Nr4a1 was regarded as homeostatic to prevent distal accumulation of spines on dendrites, thereby maintaining a normal distribution pattern of spines^23^. SNRPN overexpression resulted in a significant increase in spine density at the distal ends of dendrites. The phenotype of cortical neurons with SNRPN overexpression by *IUE* was similar to that seen in Nr4a1 knockdown *in vivo* and was rescued by expression of Nr4a1. Spines in cortical pyramidal neurons ordinarily exhibit a nonuniform distribution pattern along primary and secondary dendrites. Spine density is lowest near the soma and increases gradually to a maximum level at 40–100 mm from the soma but then decreases slightly at the most distal ends of secondary dendrites in both human and rodent brains^33^. Morphological characteristics of dendritic spines contribute to cognitive ability. The density of the spine is thought to indicate the number of excitatory synaptic inputs received by that dendrite^34^. However, the functions of spine density and distribution during development are not fully understood. Animals exhibit increased spine density after being housed in enriched environments^35^,^36^ and trained in a radial arm maze, which is proposed to improve cognition^34^. However, an abnormal increase in the number of spines and the distal accumulation of spines on dendrites were assumed to contribute to defects in the potentiation of excitatory synaptic transmission, which is associated with intellectual disabilities and ASDs.

In addition, *loss-of-function* of SNRPN leading to increased neurite length was rescued by Nr4a1 knockdown. These results confirm that SNRPN regulates neuronal morphology via Nr4a1. Furthermore, DIM has the same effects like Nr4a1 RNAi on neurite length. DIM is a Nr4a1 antagonist with a favorable safety profile and can be used for SNRPN deletion diseases, such as PWS. Nr4a1 may also be a potential drug target in diseases characterized by SNRPN.

In summary, our results reveal a novel function of SNRPN and the underlying mechanisms of SNRPN in brain development. The abnormal expression of SNRPN at embryonic stages impairs neurite outgrowth and neuronal migration. Overexpression of SNRPN disrupted the normal density and distribution of spines in apical dendrites via negative modulation of Nr4a1. We speculate that maintaining the proper level of SNRPN at different developmental stages is important in cortical neurodevelopment and that a disruption in SNRPN is linked to developmental brain disorders. Nr4a1 is a potential drug target for diseases in which SNRPN expression is abnormal, such as PWS and ASDs.

## Methods

### Constructs, antibodies and mice

Rat SNRPN (not including SNURP) was generated by PCR amplification and subcloned into the pCAG-IRES-EGFP vector with an HA tag. The Flag-Nr4a1 plasmid^23^ was provided by Dr. Yelin Chen (Interdisciplinary Research Center on Biology and Chemistry, Chinese Academy of Sciences), which was subcloned into the pCAG-IRES-EGFP vector, as well. The siRNA sequence was cloned into a pSuper vector. The sequences of SNRPN siRNAs are 5′-CAGTCGCGTTTGCGACTGG-3′ (scrambled) and 5′-GGATCGCTTACACTTGAGA-3′ (SNRPN RNAi). The sequences of Nr4a1 siRNA are refer to previously reported study^23^, 5′-CCAAGTACATCTGCCTGGCAAACAA-3′ (Nr4a1 RNAi). The efficiency of the above plasmids was verified by western blot in a primary culture of cortical neurons.

The antibodies for western blotting or immunostaining were SNRPN (rabbit, 1:500; Millipore, Billerica, MA), Nr4a1 (rabbit, 1:500; Novus, San Francisco, CA), SMI-312 (mouse, 1:1000; Covance, Princeton, NJ), green fluorescent protein (GFP) (rabbit, 1:1000; Invitrogen, Carlsbad, CA), HA (mouse, 1:1000; Covance, Princeton, NJ), and α-glyceraldehyde 3-phosphate dehydrogenase (GAPDH; mouse, 1:10000; Abcam, Cambridge, UK).

C57 Mice used in the present study were provided by SLAC Laboratory Animal Co., Ltd. (Shanghai, China). All experiments were performed in accordance with the guidelines and under the approval of the Animal Care and Use Committee of the Shanghai Institute for Biological Science of the Chinese Academy of Sciences.

### In utero electroporation (IUE)

*IUE* was performed according to previously reported methods^37^,^38^. E14.5 pregnant mice were anesthetized by intraperitoneal injection of 0.7% sodium pentobarbital (10 mL/kg). A mixture of plasmids for SNRPN cDNA (3 μg/μL) and enhanced yellow fluorescent protein (EYFP, 1 μg/μL) was injected by trans-uterus pressure microinjection into the lateral ventricle of embryos using Fast Green (2 mg/mL, Sigma-Aldrich, St. Louis, MO) as an indicator. For rescue experiments, Nr4a1 cDNA was mixed and injected with the SNRPN cDNA plasmid at a molar ratio of 1:3. Electric pulses were generated by an electroporator T830 (BTX Molecular Delivery Systems, Holliston, MA) and applied to the cerebral wall at five repeats of 30 V for 50 ms, with an interval of 1 s. In each pregnant mouse, embryos in the uterus were randomly selected to be injected with either control or test plasmids, followed by the application of electrical pulses of either the left or right direction, respectively. After internalization of the uterus, the abdominal wall was sutured in two layers. Mice were returned to their cages, given food and water ad libitum, and monitored for signs of pain or distress. After delivery, pups of the same litter were kept with their mother and euthanized at P1 and P21.

### Cell culture and transfection

Cortical tissues of E15 mice were dissected and digested by papain. Dissociated neurons were transfected with 3 μg/well of different plasmids using the Amaxa Nucleofector kit (Lonza, Basel, Switzerland) and plated into 6-well plates coated with 100 μg/mL poly-D-lysine. Cells were fed with Neurobasal medium supplemented with 10% fetal bovine serum and 2% B27. In some experiments, cells were cultured for 24 h, followed by exposure to DIM (40 μM, solved in DMSO, Santa Cruz Biotechnology, Santa Cruz, CA) for 48 h. For western blot, qPCR and immunostaining, cells were collected at DIV3 (72 h) and DIV7.

### Tissue preparation, immunostaining and imaging

Mice were transcardially perfused with 0.1 M cold PBS, followed by 4% paraformaldehyde (PFA) fixation. The brains were post-fixed for 12 h in 4% PFA and dehydrated in 15% and 30% sugar solution for 48 h at 4 °C. Coronal sections of 20 μm (for pasted sections) or 40 μm (for free-floating sections) were cut on a freezing microtome (LEICA CM1950) in six parallel sets and immediately processed for immunostaining. For cultured cells, the fixation process was 30 minutes with 4% PFA at room temperature (RT). Brain sections or cultured cells were blocked in 5% bovine serum albumin with 0.2% Triton X-100 for 1 h at RT, followed by incubation overnight at 4 degree centigrade with primary antibodies. Antibody specificity was verified by omitting the primary antibody. Fluorescently conjugated secondary antibodies were added at RT for 1.5 h. Nuclei were stained with 4′,6-Diamidino-2-phenylindole (DAPI; Sigma).

Images were acquired on a Nikon Neurolucida system for comparison of neuron distribution. The numbers of GFP^+^ neurons were counted to calculate the percentage of neurons in VZ-IZ, LoCP, and UpCP using Fuji software. For neurite length analysis, images were acquired on an Olympus FV1000 confocal system. GFP^+^ neurons were selected randomly from each condition in primary cultured cortical neurons, and the total length of all protrusions was analyzed using Fuji software. For spine density analysis, confocal z stacks of neurons in slices were acquired with the Nikon TiE-A1 plus Laser Scanning Confocal microscope, using an oil-immersion 60 × 3 objective lens. Images were analyzed with Fiji software. Protrusions in direct contact with dendrites were counted as spines, and the average spine density was calculated as the number of spines per 10 μm of dendritic length.

### RNA extraction, reverse transcription and quantitative PCR (qPCR)

Total RNA was isolated using the TRIzol reagent (Invitrogen, Carlsbad, CA) following the manufacturer’s instructions. Reverse transcription was carried out using the PrimeScript RT Master Mix (Takara). qPCRs were performed on a Rotor-Gene Q machine (QIAGEN) with SYBR Green Realtime PCR Master Mix (TOYOBO). The primers were designed according to previously reported methods^28^,^39^ and obtained as amplimer sets from Sangon Biotech (Shanghai, China). The sequences of the primers were as follows: GAPDH forward: 5′-TGACCACAGTCCATGCCATC-3′, GAPDH reverse: 5′-GACGGACACATTGGGGGTAG-3′; Nr4a1 forward: 5′-CGTTATCCGAAAGTGGGCAG-3′, Nr4a1 reverse: 5′-CGGGTTTAGATCGGTATGCCAGC-3′; and SNRPN forward: 5′-GCAAAACAGCCAGAACGTGAA-3′, SNRPN reverse: 5′-GCACACGAGCAATGCCAGTAT-3′. Data analysis was conducted by using the comparative Ct method in software by QIAGEN, and the results were normalized to GAPDH.

### Western blotting

Cortical neurons transfected with different plasmids *in vitro* and brain and lung tissues *in vivo* were prepared for western blotting. Cells or tissues were lysed in lysis buffer (RIPA, Beyotime biotechnology, Shanghai, China) with 1% Protease Inhibitor Mixture Set I (Calbiochem, San Diego, CA). Lysates were centrifuged at 12,800 g for 20 min. The supernatant was collected and denatured. Equal amounts of proteins were separated by 10% or 12% SDS-PAGE gel and blotted onto polyvinylidene difluoride membrane (Bio-Rad Laboratories, Hercules, CA). The membrane was blocked with 5% bovine serum albumin (BSA) for 1–2 h at room temperature, followed by incubation overnight at 4 °C with primary antibodies. The membrane was rinsed and then incubated for 1.5 h with peroxidase-conjugated secondary antibodies. Chemiluminescent detection was performed with the ECL kit from Pierce (Rockford, IL). Densitometric analysis was conducted using ImageJ software.

### Statistics

All data were presented as the mean ± SEM. Statistical analysis was performed using the SPSS 11.0 software. Student’s *t*-test was used to analyze statistical significance. A value of *P* < 0.05 was considered statistically significant.

## Additional Information

**How to cite this article**: Li, H. *et al*. The autism-related gene SNRPN regulates cortical and spine development via controlling nuclear receptor Nr4a1. *Sci. Rep*. **6**, 29878; doi: 10.1038/srep29878 (2016).

## Supplementary Material

Supplementary Information
