# Supplementary material for: The autism-related gene SNRPN regulates cortical and spine development via controlling nuclear receptor Nr4a1
Source: Sci Rep. 2016 Jul 19;6:29878. doi: 10.1038/srep29878 (PMC4949425; doi:10.1038/srep29878)
Supplement: Supplementary Information [file srep29878-s1.pdf]

The autism-related gene SNRPN regulates cortical and spine development via controlling nuclear receptor Nr4a1

Huiping Li[1], Pingping Zhao[2], Qiong Xu[1], Shifang Shan[2], Chunchun Hu[1], Zilong Qiu[2,*], Xiu Xu[1,*]

1 Department of Child Health Care, Children's Hospital of Fudan University, 399 Wanyuan Road, Shanghai 201102, China

2 Institute of Neuroscience, Key Laboratory of Primate Neurobiology, CAS Center for Excellence in Brain Science and Intelligence Technology, Shanghai Institutes for Biological Sciences, Chinese Academy of Sciences, Shanghai 200031, China

* Co-correspondence authors: zqiu@ion.ac.cn, xuxiu@fudan.edu.cn

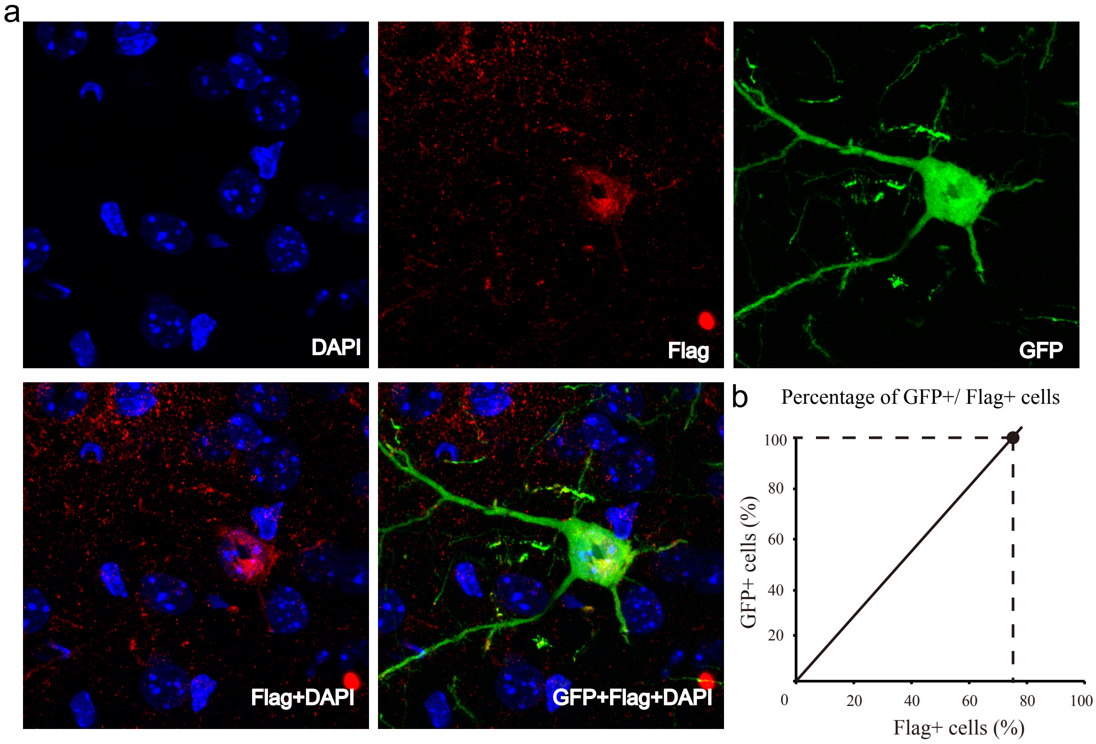

Supplementary Figure S1. Flag-tagged Nr4a1 expression in neurons co-transfection of SNRPN and Nr4a1 plasmids by *IUE*.

(a) Brain slices from P21 mice transfected with SNRPN and Nr4a1 plasmids by *IUE* were used to staining DAPI (blue), Flag (red) and GFP (green). Images showed co-localization of Flag and DAPI signals in GFP positive cells. (b) Quantification of the percentage of GFP+/ Flag+ cells in either Flag+ or GFP+ cells.
